# Perinatal Exposure to the Neonicotinoid Thiacloprid Impacts Transcription of Neuroplasticity and Neuroendocrine Markers in Mice but Not in the Zebrafish Model

**DOI:** 10.1002/jat.4878

**Published:** 2025-08-03

**Authors:** Kirthana Kunikullaya U, Zuzanna M. Baran, Pascal Coumailleau, Laetitia Guillot, Harry W. M. Steinbusch, Fatima Smagulova, Thierry D. Charlier

**Affiliations:** ^1^ Inserm, EHESP, Irset (Institut de Recherche en Santé, Environnement et Travail) University of Rennes Rennes France; ^2^ Department of Physiology, Sri Chamundeshwari Medical College Hospital and Research Institute Channapatna Karnataka India; ^3^ Simons Initiative for the Developing Brain University of Edinburgh Edinburgh UK; ^4^ Psychiatry and Neuropsychology, School for Mental Health and Neuroscience (MHeNs) Maastricht University Maastricht the Netherlands; ^5^ ImPACcell Platform, UAR Biosit, CNRS 3480 Inserm 01 University of Rennes Rennes France

**Keywords:** amygdala, cerebellum, hippocampus, hypothalamus, mouse, neonicotinoid, neurogenesis, neuroplasticity, thiacloprid, zebrafish

## Abstract

Neonicotinoids are widely used insecticides in agriculture, aquaculture, pet care, and urban pest control. Initially developed to selectively target the insect cholinergic system, their extensive use has raised concerns about adverse effects on nontarget vertebrates. This study investigated the developmental neurotoxicity of the neonicotinoid thiacloprid using two vertebrate models: zebrafish and mice. Transgenic *cyp19a1b‐GFP* zebrafish eleutheroembryos, which report estrogenic activity, were exposed to thiacloprid (10^−6^–10^−8^ M) for 4–5 days. No significant changes were observed in GFP expression or neuroplasticity and neuroendocrine markers, suggesting a limited impact in this aquatic model. In contrast, prenatal exposure of mice to thiacloprid (0.06, 0.6, or 6 mg/kg/day from embryonic day 6.5 to 15.5) produced dose‐, sex‐, and region‐specific alterations in brain gene expression during adolescence (postnatal day 35). At low to mid doses, markers of neurogenesis and plasticity, such as doublecortin in the amygdala, neurogenin, nestin, and *PCNA* in the hippocampus and cerebellum, were upregulated. However, high‐dose exposure (6 mg/kg/day) led to reduced expression of these markers, including *BDNF* in the hypothalamus and *PCNA* in the hippocampus, particularly in females. These results indicate that thiacloprid, even at low doses, can subtly but significantly affect mammalian brain development. Further research is needed to assess the neurodevelopmental risks of neonicotinoids in vertebrates, including humans.

## Abstract 2

Neonicotinoids, widely used insecticides, may pose risks to nontarget vertebrates. This study assessed thiacloprid's developmental neurotoxicity using zebrafish and mice. Zebrafish exposed to low concentrations showed no significant neurobiological effects. In contrast, prenatal thiacloprid exposure in mice caused sex‐ and region‐specific brain gene expression changes. Low to mid doses increased markers of neurogenesis and neuroplasticity, while the highest dose reduced these markers, especially in females. Results suggest dose‐dependent neurodevelopmental effects of thiacloprid in vertebrates.

## 1 Introduction

Neonicotinoids are insecticides commonly used in agriculture, aquaculture (fish farming), pet treatment, and urban pest control. These are structurally related to nicotine and target nicotinic cholinergic receptors (nAChRs), the membrane receptors sensitive to the neurotransmitter acetylcholine (ACh) (Simon‐Delso et al. 2015). Each neonicotinoid exhibits distinct binding characteristics to the nAChRs (Lu et al. 2022; Tomizawa and Casida 2000), and likely, the specificity of these subunits is also species‐dependent. Neonicotinoids harness the existing structural differences between invertebrate and vertebrate nAChRs with a very strong affinity for insect receptors while exhibiting a much lower affinity to vertebrate subunits (for review, see Tomizawa and Casida 2000; Tomizawa et al. 2000; Jeschke et al. 2011; Houchat et al. 2020). The nicotinic receptors are functionally present in vertebrates as homo‐ or hetero‐pentameric receptors, a combination of alpha (α1 to α9) and nonalpha subunits (β1 to β4, δ, ε, or γ), at the neuromuscular junction and in the central and peripheral nervous system. In mammals, neonicotinoids are shown to act on the α4β2, α3β4, and α7 types of nAChRs (Li et al. 2011; Ramachandran Nair and Liu 2019; Xiang et al. 2020; Hirano et al. 2019). These receptors are expressed very early during the development of mammals, and the combination of subunits and the regional patterning varies during pre‐ and postnatal development (see, e.g., Hellström‐Lindahl et al. 1998; Rima et al. 2020; Alzu'bi et al. 2020; Broide et al. 2019; Arenzana et al. 2005). Neonicotinoids are far less toxic to the handlers and nontarget organisms in comparison to other insecticides such as organophosphate and carbamate: the geometric mean of lethal dose 50% (LD50) in rats from eight neonicotinoids is 912 mg/kg bodyweight (bw) for neonicotinoids via acute oral exposure, ranging from 182 for acetamiprid to > 5000 mg/kg, 600 mg/kg for thiacloprid, while the geometric mean, based on very large data sets, was 67 and 45 mg/kg bw for organophosphate and carbamate, respectively (Tomizawa and Casida 2000, 2005). However, the intensive use of neonicotinoids and the persistence of the molecules in the environment contribute to the increased exposure of nontarget invertebrates, more particularly honeybees and other pollinating insects, and vertebrates (Anadón et al. 2020; Rundlöf et al. 2015). Indeed, due to systemic distribution throughout the plant (Simon‐Delso et al. 2015; Ssemugabo et al. 2022), the molecule is found in fruits and vegetables (Ssemugabo et al. 2022; Chen et al. 2014, 2022; García‐Valcárcel et al. 2022; Li et al. 2020; Abdelfatah et al. 2020). A few studies suggest that several neonicotinoids, including imidacloprid, acetamiprid, and thiacloprid, can readily cross the intestinal barrier (Brunet et al. 2004; Chedik et al. 2017) and the blood–brain barrier (Chedik et al. 2017; Campbell et al. 2022; Passoni et al. 2021; Terayama et al. 2016; Sheets et al. 2016), and these pesticides and their metabolites are found in human biological samples, confirming human exposure (Harada et al. 2016; Laubscher et al. 2022; Li and Kannan 2020; Oya et al. 2021; Pan et al. 2022; Wrobel et al. 2022; Ichikawa et al. 2019). Due to chronic exposure to neonicotinoids and their potential bioavailability in mammalian organisms, questions and concerns were raised about potential adverse health effects in humans, including cancers, neurodevelopmental disorders, and other pathologies (Thompson et al. 2020).

Many studies highlight the impact of neonicotinoids such as acetamiprid, imidacloprid, and clothianidin on vertebrate locomotor activity and behavior via a direct impact on the nervous system. For example, imidacloprid and clothianidin were shown to significantly affect locomotor activity and emotion‐like behavior during behavioral tests in mice and rats. These effects are context‐dependent and may include reduced exploration, anxiety‐like responses, and/or cognitive impairment (Tonietto et al. 2022; Burke et al. 2018; Abd‐Elhakim et al. 2018; Tanaka 2012, 2021; Hirano et al. 2018). Interestingly, the opposite effect on locomotor activity was observed in aquatic vertebrate species, including amphibians (Lee‐Jenkins and Robinson 2018; Holtswarth et al. 2019) and zebrafish (Crosby et al. 2015; Guerra et al. 2021; Könemann et al. 2022). In addition to locomotion, learning and memory were also affected in rodents following exposure to some neonicotinoids (Mora‐Gutiérrez et al. 2021; Kara et al. 2015; Shamsi et al. 2021; Tasman et al. 2021; Gross 2013; Akkoc et al. 2020). These behavioral alterations are linked to the impact on neurons and neurotransmission in the peripheral and/or central nervous system, as shown by in vitro and in vivo studies (Nakayama et al. 2019; Kimura‐Kuroda et al. 2012; Maeda et al. 2021; Loser et al. 2021; Cimino et al. 2017; Faro et al. 2012). These alterations could be linked to the activation of central nAChRs as the cholinergic system situated in the basal forebrain and brainstem innervates the entire central nervous system (Gotti et al. 2006; Holgate and Bartlett 2015). It should also be noted that more recent studies report the potential endocrine‐disrupting action of neonicotinoid as suggested by a decline in fertility rate (Abdel‐Rahman Mohamed et al. 2017; Mikolić and Karačonji 2018; Hartman et al. 2021), impact on steroidogenic enzymes such as aromatase (Caron‐Beaudoin et al. 2016, 2017, 2018), and changes in circulating sex hormones, including follicle stimulating hormone (FSH), estrogens, and testosterone (Kapoor et al. 2011; Kong et al. 2017; Schmidt 2018). The brain itself is a major steroidogenic site, and neurosteroidogenesis is fundamental for brain development and physiology (for reviews, see Tsutsui 2012; Schlinger and Remage‐Healey 2012; Charlier et al. 2015; Diotel et al. 2018; Brann et al. 2021). Any change in brain steroid synthesis and bioavailability during development, including endocrine disruptor exposure, leads to significant long‐term defects in brain plasticity and behavior (see, e.g., Brann et al. 2022; McCarthy 2020; Reddy et al. 2022; Takesono et al. 2022; Kight and McCarthy 2020).

While the majority of studies on neonicotinoids focused on the impact of imidacloprid, acetamiprid, or clothianidin on the brain and the endocrine system, far less is known about the potential long‐term effect of early exposure to thiacloprid [(Z)‐thiacloprid, (3‐((6‐chloro‐3‐pyridinyl)methyl)‐2‐thiazolidinylidene)cyanamide] (PubChem n.d.). Thiacloprid is another widely used neonicotinoid whose renewal was rejected on February 3, 2020, in Europe, but repeated emergency authorizations for use in sugar beets and berries are permitted (see Authority [EFSA] EFS et al. 2019). Thiacloprid shows a similar mode of action as the other neonicotinoids, although its LC50 (lethal concentration 50, the concentration that kills 50% of the animals) is slightly lower in various aquatic invertebrates (Morrissey et al. 2015). Toxicity for vertebrates varies depending on the species and their habitat. It is usually lower for aquatic vertebrates (LC50 29.6 mg/L in rainbow trout and 24.5 mg/L in bluegill sunfish) and higher in terrestrial vertebrates (> 200 mg/kg bw in mallard ducks and chicken) and sexually differentiated in rats (836 mg/kg bw in males; 444 mg/kg in females) (FAO Specifications and Evaluations for Agricultural Pesticides—Thiacloprid [Internet] 2010). The ‘No observed adverse effect level’ (NOAEL) for thiacloprid is currently set at 1.2 mg/kg bw per day based on liver histopathology and eye effects resulting from a chronic, 2‐year, oral exposure study performed in rats (European Food Safety Authority [EFSA] et al. 2019).

This study aimed to investigate the impact of early thiacloprid exposure on neuroplasticity, including neurogenesis and synaptic changes, and link these effects to potential changes with local steroid action in the brain. The potential differences between aquatic and terrestrial vertebrates were also considered by studying the impact of thiacloprid exposure on zebrafish and mice, respectively. Based on previous studies and current accepted regulatory limits for thiacloprid, we chose concentrations (10^−6^–10^−8^ M for zebrafish) and doses (0.06–6 mg/kg bw for mouse) around and below the NOAEL for short‐term exposure. The goal was also to study potential sex differences in mice, as many previous studies investigating the long‐term impact of neonicotinoids on the brain were performed only on males (see Abou‐Donia et al. 2008) while brain neuroplasticity is often sexually differentiated (Uhl et al. 2022; DeCasien et al. 2022; Bakker 2022). Moreover, recent studies indicate that certain neonicotinoids, such as clothianidin, affect behavioral traits differently depending on sex, with either male (Kubo et al. 2022) or female (Kaku et al. 2024) showing an increased sensitivity.

## 2 Materials and Methods

### 2.1 Animals

Zebrafish (Experiments 1a and 1b) and mice (Experiments 2a and 2b) were handled and euthanized in agreement with the guidelines for the use and care of laboratory animals and in compliance with French and European regulations on animal welfare. The animal facilities used for the present study are licensed by the French Ministry of Agriculture (zebrafish: Biosit ARCHE: agreement number B35‐238‐40 and mice: IRSET agreement number D35–238–19). All animal procedures were performed according to the Ethics Committee of the Ministry of Research of France (agreement number: 17473‐2018110914399411). All experimental procedures followed the ethical principles outlined in the Ministry of Research Guide for Care and Use of Laboratory Animals and were approved by the local Animal Experimentation Ethics Committee (C2EA‐07).

### 2.2 Experiment 1: Zebrafish Eleutheroembryo Exposure to Thiacloprid

**FIGURE 1:**
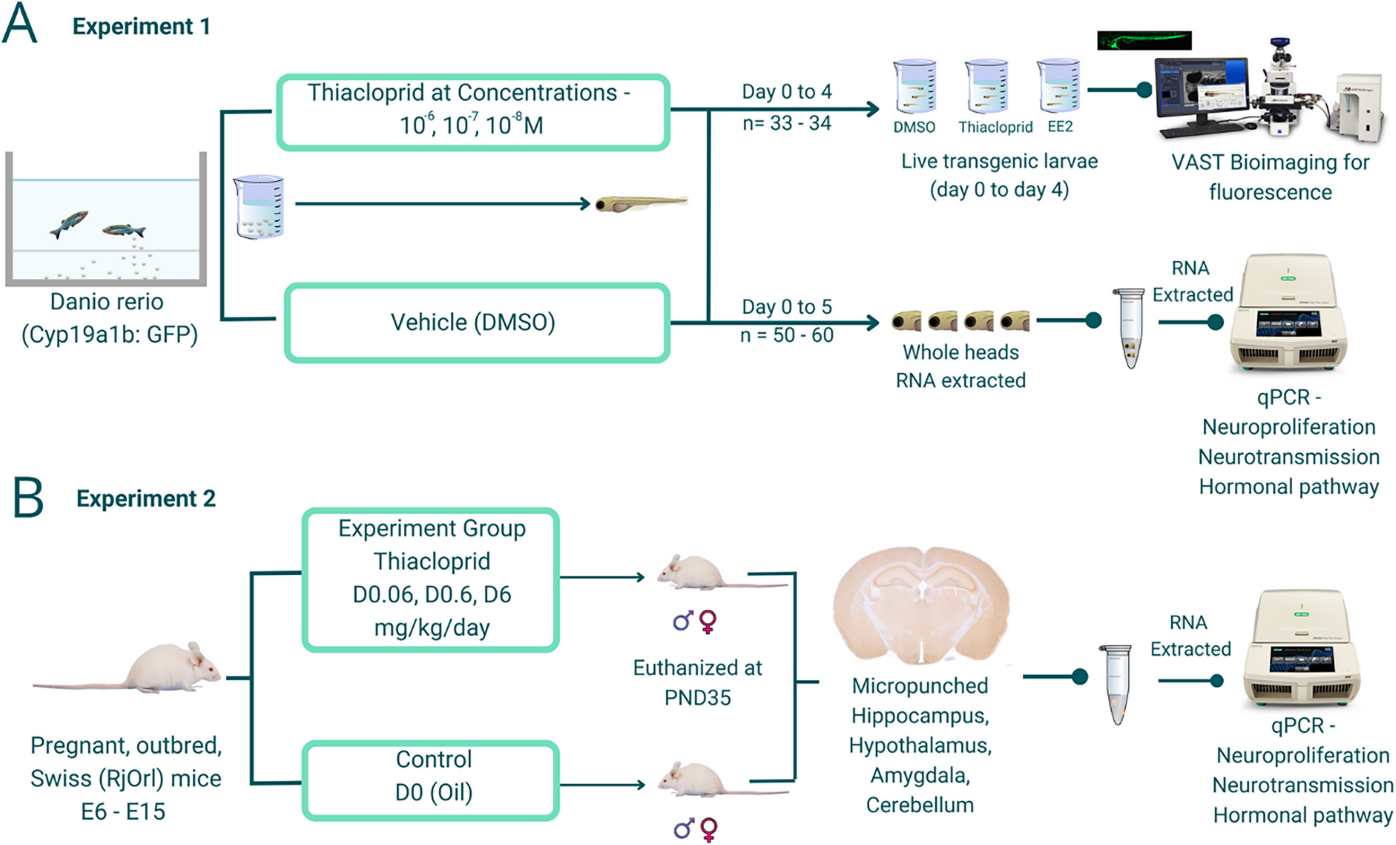
Experimental design of the study. Protocol followed for (A) zebrafish (Experiment 1) and (B) mice (Experiments 2a and 2b). In Experiment 2a—D0 = control group and D6 = 6 mg/kg/day thiacloprid, oral gavage. In Experiment 2b—D0 = control, D0.06 = 0.06 mg/kg/day thiacloprid, D0.6 = 0.6 mg/kg/day thiacloprid.

We used adult transgenic zebrafish tg (cyp19a1b‐GFP) (90 dpf), expressing GFP (green fluorescent protein) under the control of the brain aromatase *cyp19a1b* gene promoter (Tong et al. 2009). *Cyp19a1b* encodes the estrogen‐dependent brain aromatase specifically expressed in radial glial cells in the fish brain. This specific line is used in the The Organisation for Economic Co‐operation and Development (OECD) guideline test 250 — Detection of Endocrine Active Substances, acting through estrogen receptors, using transgenic tg(cyp19a1b:GFP) Zebrafish embrYos (EASZY) assay (OECD 2021)—and is shown to be a valuable assay to test the estrogenic activity of various xenobiotic compounds (Cano‐Nicolau et al. 2016). Adult fish were housed in our facility (ImPACcell, BIOSIT) in a recirculation system (Zebtec, Tecniplast, Italy) under standard conditions of photoperiod (14 h light and 10 h dark) and temperature (28°C ± 1°C) with a 20% daily water change. Fish were fed twice daily with dry food (Gemma Micro ZF, Planktovie SAS). Fish were treated as described in the schema for 6 days and used doses indicated (Figure 1A).

#### 2.2.1 Experiment 1a: EASZY Assay

Eggs obtained from two independent mass spawning (11 and 17 adult males and females in two aquaria) were collected immediately after spawning and grown in E3 medium (5 mM NaCl, 0.17 mM KCl, 0.33 mM CaCl2, and 0.33 mM MgSO4 in distilled water) at 28°C in glass Petri dishes. Four hours post‐fertilization (hpf), developing embryos were randomly distributed into five groups of approximately 100 eggs: Three groups were exposed to a final concentration of 10^−6^, 10^−7^, or 10^−8^ M thiacloprid dissolved in dimethyl sulfoxide (DMSO)  (total *n* = 32 in each treatment group from two independent experiments), one group exposed to 0.5 × 10^−10^ M (0.05 nM) ethinylestradiol (EE2, used as positive control in the EASZY assay, total *n* = 34 from two independent experiments) and the control group was exposed to only DMSO (total *n* = 33 from two independent experiments). The maximum volume of the solvent did not exceed 0.01% (v/v) (5 μL in 50 mL E3). The exposure medium was changed every day for 4 days, following OECD 250 guidelines. Following exposure, eleutheroembryos were anesthetized using Tricaine methanesulfonate or MS222 (150 μg/mL). Automated imaging of zebrafish developmental phenotypes was conducted with the VAST BioImager (Union Biometrica Gees, Belgium), a system that allows the automatic positioning of zebrafish embryos and eleutheroembryos (Pardo‐Martin et al. 2010). Each live tg (cyp19a1b‐GFP) transgenic eleutheroembryo was loaded individually via the hand‐held flow‐through pipettor, correctly oriented with the VAST BioImager, and was photographed once in dorsal and once in lateral view using a Zeiss AxioImager M1 fluorescence microscope equipped with an AxioCam 506 camera (Zeiss GmbH, Göttingen, Germany). The same exposure conditions were used to acquire each photograph (×10 objective, 70 ms of fluorescent light exposure, maximal light intensity). Fluorescence quantification was performed using Fiji software and FAST plugin (ImageJ2 v.2.14.0/1.54f; available online: http://rsb.info.nih.gov/ij/; Schindelin et al. 2012) based on previous protocols (Brion et al. 2019). For each picture, the intensity of fluorescence was measured through the integrated density (IntDen), that is, the sum of the gray values of all the pixels within the region of interest. Gray values of 300 or below were considered background values.

#### 2.2.2 Experiment 1b: Transcription Pattern

Zebrafish eggs were collected immediately after mass spawning of the transgenic tg (cyp19a1b‐GFP) zebrafish and grown in E3 medium at 28°C in glass Petri dishes. Developing embryos were randomly distributed within 4 hpf into four groups of approximately 100 eggs: Three groups were exposed to 10^−6^, 10^−7^, or 10^−8^ M thiacloprid dissolved in DMSO, and the control group was exposed to DMSO only (4 μL in 40 mL E3). The medium was changed every day for 5 days. Mortality in the embryos and any other morphological abnormalities were observed over this duration. On day 6, 50–60 eleutheroembryos per group were terminally anesthetized with MS222 (1 mg/mL). Whole heads were collected, immediately frozen in liquid nitrogen, and stored at −80°C before RNA extraction and quantitative real‐time PCR. This protocol was repeated seven times such that each experimental exposure represents one biological sample, and the final number of biological samples is 7 (*n* = 7). Each sample was sonicated for 15 s in 250 μL of NucleoZol reagent (Macherey‐Nagel), and RNA extractions were performed using the NucleoSpin RNA Plus kit (Macherey‐Nagel) following the manufacturer's instructions.

### 2.3 Experiment 2: Prenatal Exposure to Thiacloprid in Mice

#### 2.3.1 Mice Treatment and Dissection

Outbred Swiss mice (RjOrl) were purchased from Janvier, France, and acclimatized in our facilities for 1 week before random assignment to the groups. Animals were kept under standard laboratory conditions in a 12:12‐h light/dark schedule with access to standard mouse chow and tap water ad libitum. Females were then bred, and the vaginal plug was checked in the morning. The day of the vaginal plug was considered embryonic day 0.5 (E0.5), and pregnant females were placed in an individual cage. From E6.5 until E15.5, these mice were treated with 6 mg/kg/day (Experiment 2a), 0.6 or 0.06 mg/kg/day (Experiment 2b) thiacloprid suspended in olive oil via oral gavage (150 μL; see Hartman et al. 2021) or only olive oil (control/D0). For each dose, a minimum of four unrelated pregnant mice were treated. The 6 mg/kg/day is a dose just around the NOAEL for mice and rats in developmental neurotoxicity and carcinogenicity studies (Authority [EFSA] EFS et al. 2019). The male and female progeny were weaned on the 21^st^ day, and four siblings of the same litter were housed per cage. F1 generation male and female mice (maximum two per litter) were euthanized at the age of 35 days (postnatal day PND 35) after blood collection from the retro‐orbital vein. This exposure protocol and the timing of exposure and euthanasia were initially developed to investigate the effects of thiacloprid on testicular development during adolescence (Hartman et al. 2021). In addition, PND 35 is a critical time point in mouse development that corresponds to preadolescence in humans and is a period of significant brain maturation and synaptic remodeling (Semple et al. 2013). The brains were dissected and placed immediately on dried ice and stored at −80°C until use. Brains were cut into 300‐μm‐thick sections with a cryostat (Microm HM560), and bilateral punches were collected using the Stoelting brain punch set (diameter 1.25 mm) from three areas of interest: hypothalamus, hippocampus, and amygdala. The cerebellum was also collected and analyzed in Experiment 2b. The number of brain samples collected for each area was 15 for controls (7 males and 8 females) and 16 for the 6 mg/kg/day treatment group (10 males and 6 females) in Experiment 2a and 10 for controls (5 males and 5 females), 8 for 0.6 mg/kg/day (4 males and 4 females), and 8 for 0.06 mg/kg/day (4 males and 4 females) in Experiment 2b. Total RNA was extracted using the NucleoSpin kit for Nucleozol (Macherey‐Nagel), and quantity and quality were determined on NanoDrop 8000 Spectrophotometer (Thermo Fishcher Scientific).

#### 2.3.2 RT‐qPCR

RNA (1 μg) from zebrafish and mice was reverse transcribed using Moloney Murine Leukemia Virus Reverse Transcriptase (MMLV‐RT) (Promega) following the manufacturer's protocol and using random primers. Quantitative polymerase chain reaction (qPCR) was performed using Sybr Green (iTaq SYBR, Biorad). Markers of cell proliferation (Proliferative cell nuclear antigen/*pcna/Pcna*), neuronal differentiation (Nestin/*nes/Nes*, Neurogenin/*neurog1/Neurog1*, doublecortin/*Dcx*), neuronal markers (Brain derived neurotrophic factor/*bdnf/Bdnf for mouse only*, Synaptophysin/*sypb/Syp*, Synapsin IIa/*syn2a/Syn2a*), and neuroendocrine‐linked proteins (estrogen receptors alpha/*esr1/Esr1*, beta/*Esr2 for mouse or* beta1/*esr2b and* beta2/*esr2a for zebrafish*, aromatase/*cyp19a1b/Cyp19a1*) were analyzed. Activated caspase 3 (apoptosis‐related cysteine peptidase a [*casp3a*]) was tested for zebrafish only. Housekeeping genes used were E74‐like ETS transcription factor 1 (*elf1)* for zebrafish and glyceraldehyde‐3‐phosphate dehydrogenase (GAPDH) for the mouse. The threshold cycle (Ct) was determined for each gene, and a melting curve was obtained for each sample to confirm specificity. Relative gene expressions were calculated using the 2^−ΔΔCt^ method for relative quantification (Schmittgen and Livak 2008). The induction or inhibition was determined and expressed as a fold change compared with the normalized control condition (the male control group in mice experiments). Primer sets used for qPCR are presented in Table S1.

### 2.4 Statistical Analysis

Data are represented as the mean ± standard error of the mean (SEM). Outliers, defined as values outside the mean ± 2 standard deviations, were removed from the analysis (the number of animals remaining is plotted in the graphs). The treatment effect was analyzed with a one‐way analysis of variance (ANOVA) for Experiment 1 (zebrafish) and a two‐way ANOVA for Experiment 2 (mice) with sex and dose as factors for each brain region (Statistica Version 13, Dell Inc.). Post hoc analysis was performed using the Tukey post hoc test where appropriate. The values were considered statistically significant if *p* < 0.05. The figures were generated using GraphPad Prism (Version 9).

## 3 Results

### 3.1 Experiment 1: Zebrafish Exposure

#### 3.1.1 Experiment 1a: EASZY Assay

**FIGURE 2:**
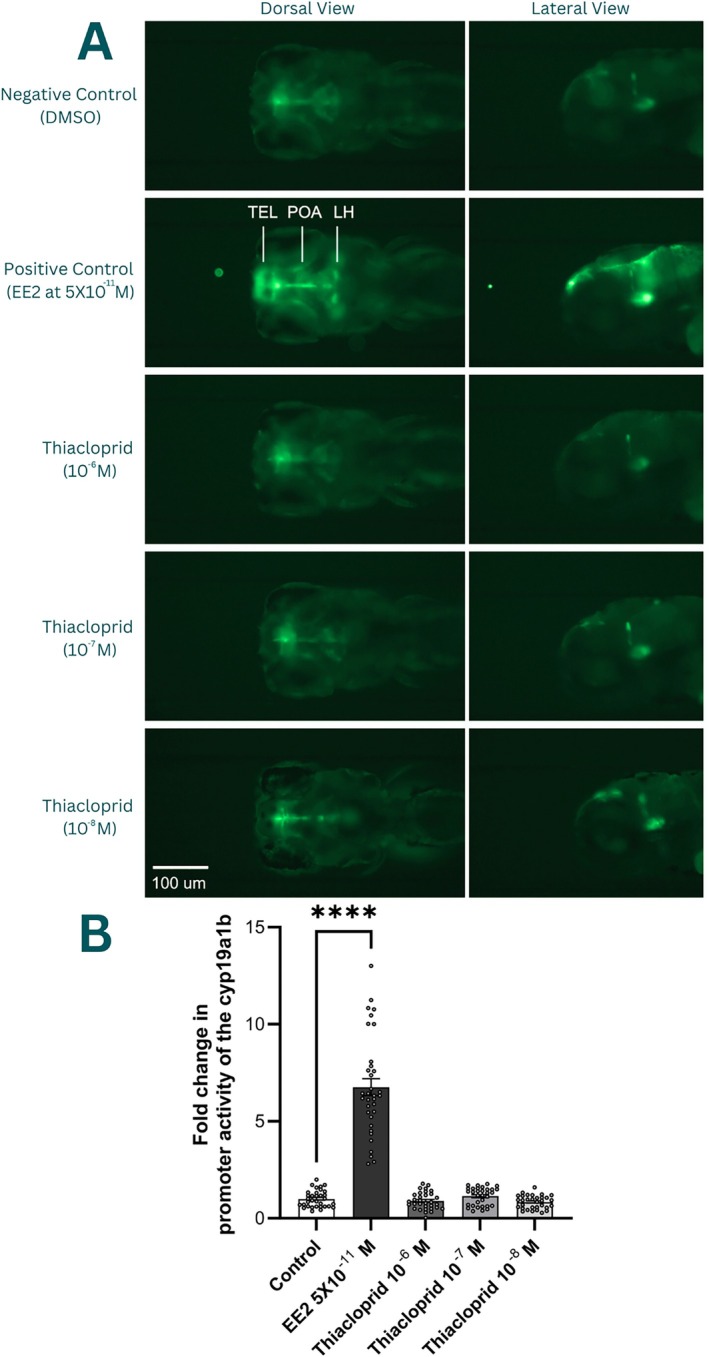
The effects of thiacloprid on GFP expression reflecting estrogen‐dependent cyp19a1b promoter activity in larval zebrafish; (A**)** representative images of zebrafish head showing the difference in fluorescence intensity due to the promoter activity of the cyp19a1b in control (DMSO), positive control (EE2 at 5 × 10^−11^ M), thiacloprid at 10^−6^, 10^−7^, and 10^−8^ M, with regions of high promoter activity indicated in the positive control—TEL, telencephalon; POA, preoptic area; LH, lateral hypothalamus; (B) quantification of the fold change in fluorescence integrated density, ****p < 0.05 versus control group.

The quantification of fluorescence in the brain, reflecting the promoter activity of the brain aromatase (*cyp19a1b*‐GFP) revealed a strong and statistically significant impact of the treatment *F*(4, 158) = 149.2, driven by the significant increase in the positive control, EE2 (*p* > 0.0001 Dunnett's test versus control group), as expected. The concentration used was 0.05 nM, indicating the very high sensitivity of the assay. Thiacloprid, independent of the concentration used, did not affect the fluorescence intensity in the radial glial cells, as compared with the control condition, indicating an absence of estrogenic activity in our experimental condition (Figure 2).

#### 3.1.2 Experiment 1b: Transcription Pattern

**FIGURE 3:**
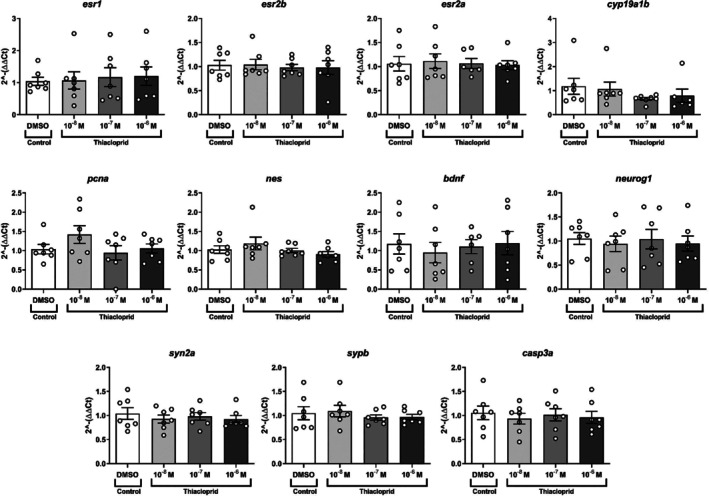
Mean (± SEM) fold change (2^−∆∆Ct^ values) and individual transcription levels of 11 genes in zebrafish eleutheroembryo heads following 5 days of exposure to three different concentrations of thiacloprid (10^−8^, 10^−7^, 10^−6^ M). No statistically significant difference was observed. Each dot represents one experimental point (*n* = 7 independent experiments), each containing a pool of 50–60 heads. Proliferative cell nuclear antigen/*pcna*, Nestin/*nes*, Neurogenin/*neurog1*, Brain‐derived neurotrophic factor/*bdnf*, Synaptophysin/*sypb*, Synapsin IIa/*syn2a*, estrogen receptors alpha/*esr1*, beta1/*esr2b*, beta2/*esr2a*, aromatase/*cyp19a1b*, Activated caspase 3/apoptosis‐related cysteine peptidase a (*casp3a*).

In fish, developmental exposure to three concentrations of thiacloprid (10^−8^, 10^−7^, 10^−6^ M) for 5 days did not impact the developmental mortality, nor were any other abnormalities noted in the embryos (data not shown). In addition, none of the three different concentrations of thiacloprid impacted the transcription of any of the markers used in the experimental conditions when compared with control samples (*p* > 0.05; Figure 3).

### 3.2 Experiment 2: Mouse Exposure

#### 3.2.1 Experiment 2a: 6 mg/kg/day Thiacloprid

The first analysis was performed to determine whether in utero exposure to 6 mg/kg/day thiacloprid would affect neuroendocrine and neuroplasticity markers in adolescent (PND 35) male and female mice offsprings. Three regions of interest were investigated: the amygdala, the hypothalamus, and the hippocampus, and the transcription levels of 10 genes were quantified.

##### 3.2.1.1 Amygdala

**FIGURE 4:**
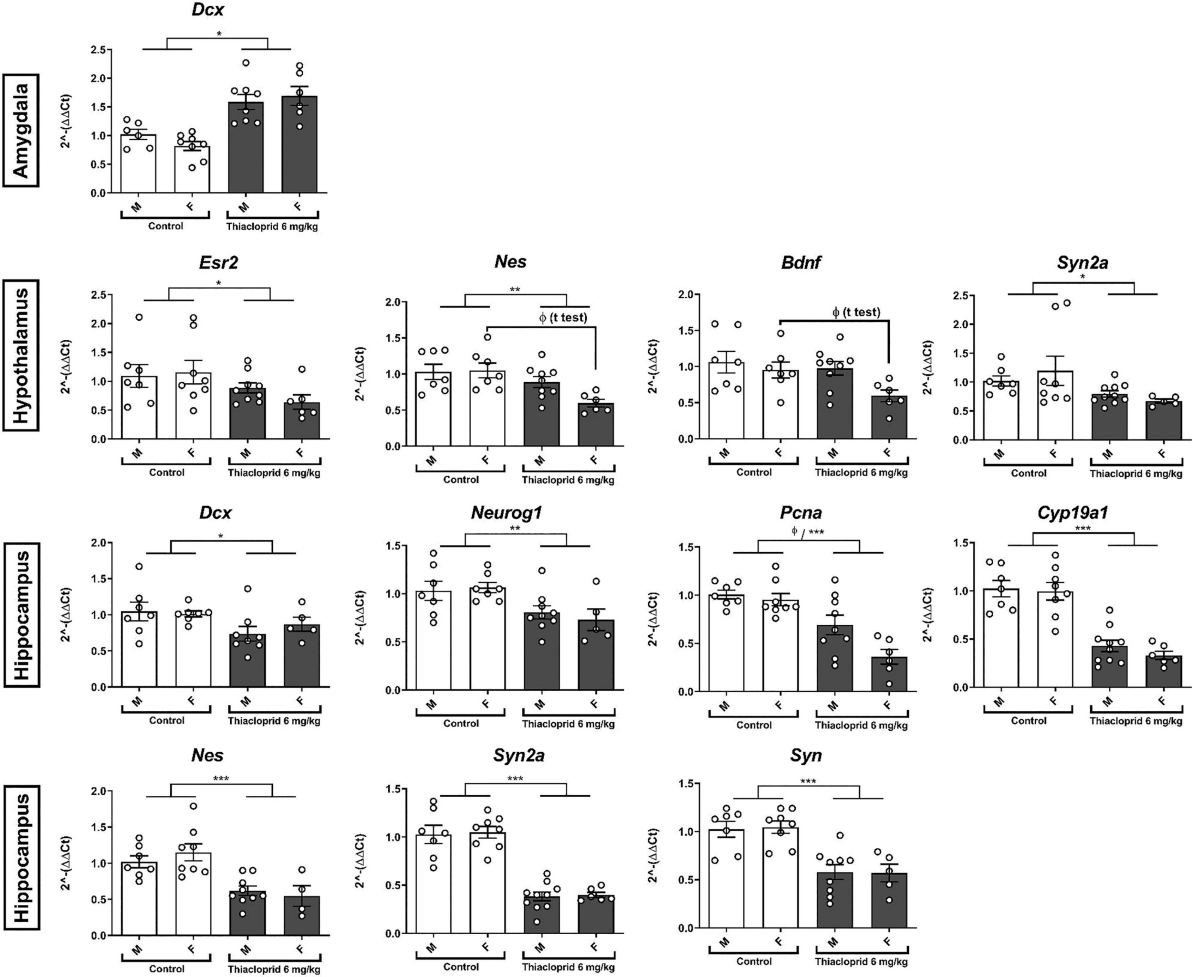
Mean (± SEM) fold change (2^−∆∆Ct^ values) and individual transcription levels of a selected subset of genes in the amygdala, hippocampus, and hypothalamus in male (M) and female (F) mice offspring (PND 35) following in utero exposure to thiacloprid (6 mg/kg/day); ***p < 0.001, **p < 0.01, *p < 0.05; ɸ significant reduction in female mice (a posteriori analysis). Proliferating cell nuclear antigen*/Pcna*, doublecortin/*Dcx*, nestin/*Nes*, neurogenin/*Neurog1*, brain‐derived neurotrophic factor/*Bdnf*, synaptophysin/*Syp*, and synapsin IIa/*Syn2a*, and estrogen receptors alpha/*Esr1*, beta/*Esr2*, aromatase/*Cyp19a1*.

A significant main effect of treatment on *Dcx* transcription (*F*(1, 24) = 35.806, *p* < 0.0001) with an increase after 6 mg/kg/day thiacloprid exposure, but no effect of sex and no interaction between treatment and sex was observed (Figure 4). There was no other significant effect of treatment and sex nor any interaction for the other markers (see Table S2 for the statistical results for all 10 genes and Table S3 for Tukey's post hoc test results).

##### 3.2.1.2 Hypothalamus

A statistically significant effect of thiacloprid exposure on *Esr2* (*F*(1, 26) = 5.041, *p* = 0.033), nestin (*F*(1, 25) = 11.339, *p* = 0.002), and synapsin IIa (*F*(1, 26) = 6.021, *p* = 0.021) transcription in the hypothalamus was found. It can be noted that there was a trend toward an interaction between sex and treatment on nestin transcription (*F*(1, 25) = 3.047, *p* = 0.09), where the mean fold change was reduced in thiacloprid‐exposed females compared with the other groups. A significant effect of sex on *Bdnf* transcription (*F*(1, 25) = 4.6810, *p* = 0.040) with females exhibiting a reduction compared with males and a tendency toward the main effect of treatment (*F*(1, 25) = 3.744, *p* = 0.064) was also found (Figure 4) but no interaction between treatment and sex, although females seemed to be mostly affected. Indeed, a posteriori t‐tests performed in females only showed a trending reduction of *Esr2* (*p* = 0.00698), synapsin IIA (*p* = 0.054), and a significant reduction of nestin (*p* = 0.0032) and *Bdnf* (*p* = 0.0277) in the experimental group versus the control females. No other statistically significant difference was observed in the hypothalamus (see Table S2 for the statistical results for all 10 genes and Table S3 for Tukey's post hoc test results).

##### 3.2.1.3 Hippocampus

A significant main effect of thiacloprid exposure, with a reduction of *Dcx* (*F*(1, 23) = 4.988, *p* = 0.036), aromatase (*F*(1, 27) = 68.360, *p* < 0.0001), neurogenin (*F*(1, 24) = 10.903, *p* = 0.003), nestin (*F*(1, 24) = 23.649, *p* < 0.0001), synapsin IIa (*F*(1, 27) = 106.908, *p* < 0.0001), synaptophysin (*F*(1, 25) = 32.413, *p* < 0.0001), and *Pcna* (*F*(1, 26) = 31.671, *p* < 0.0001) transcription, was observed. *Pcna* transcription was impacted by sex (*F*(1, 26) = 5.643, *p* = 0.025) with a reduction in females compared with males, but no interaction between the two factors. There was a trend toward an interaction between sex and treatment on *Pcna* transcription (*F*(1, 26) = 3.008, *p* = 0.095), where the mean fold change was reduced in thiacloprid‐exposed females compared with the other groups. Furthermore, a posteriori t‐tests performed in females only showed a significant reduction of *Pcna* (*p* < 0.0001) in the experimental group versus the control females (Figure 4). No other difference was observed in the hippocampus (see Table S2 for the statistical results for all 10 genes and Table S3 for Tukey's post hoc test results).

#### 3.2.2 Experiment 2b

The impact of lower doses of thiacloprid (0.6 and 0.06 mg/kg/day) on the same 10 neuroplasticity and neuroendocrine markers was next investigated in the amygdala, hypothalamus, hippocampus, as well as in the cerebellum.

##### 3.2.2.1 Amygdala

**FIGURE 5:**
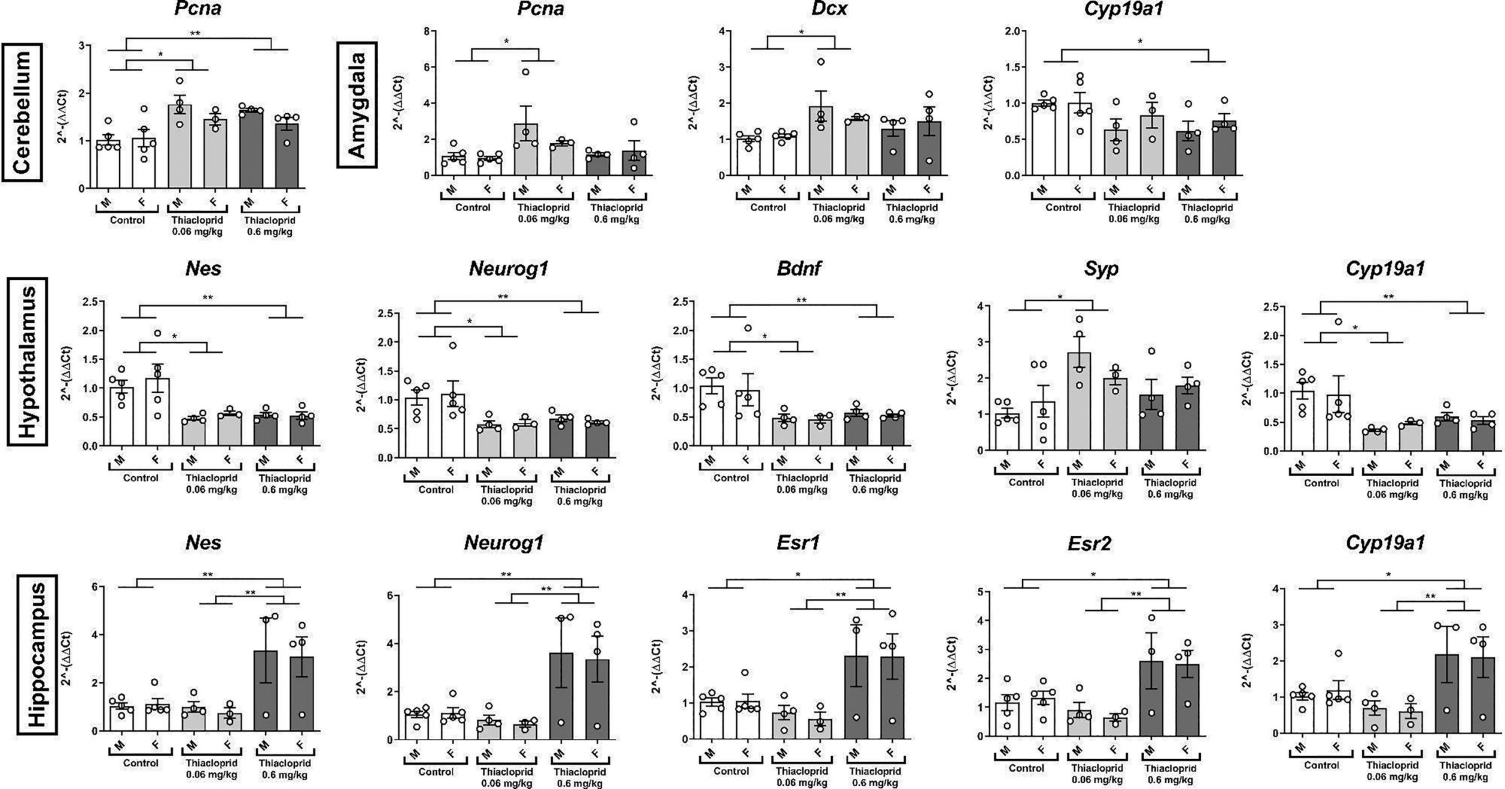
Mean (± SEM) fold change (2^−∆∆Ct^ values) and individual transcription levels of markers in the cerebellum, amygdala, hypothalamus, and hippocampus in male (M) and female (F) mice offspring (PND35) following in utero exposure to thiacloprid (0.06 and 0.6 mg/kg/day) in Experiment 2b; ***p < 0.001 **p < 0.01 *p < 0.05. Proliferating cell nuclear antigen*/Pcna*, doublecortin/*Dcx*, nestin/*Nes*, neurogenin/*Neurog1*, brain‐derived neurotrophic factor/*Bdnf*, synaptophysin/*Syp*, and estrogen receptors alpha/*Esr1*, beta/*Esr2*, aromatase/*Cyp19a1*.

A statistically significant effect of thiacloprid on *Dcx* (*F*(2, 19) = 4.065, *p* = 0.034), *Pcna* (*F*(2, 19) = 4.441, *p* = 0.026), and aromatase (*F*(2, 19) = 4.116, *p* = 0.033). Post hoc analysis showed that the lowest dose of 0.06 mg/kg/day significantly increased *Dcx* (*p* = 0.021) and *Pcna* (*p* = 0.016) transcription compared with the control group, while the dose of 0.6 mg/kg/day significantly reduced aromatase transcription in comparison to the control group (*p* = 0.042) (Figure 5). There was no sex difference or interaction between treatment and sex. No other difference was observed for the other transcripts in the amygdala (see Table S4 for the statistical results for all 10 genes and Table S5 for Tukey's post hoc test results).

##### 3.2.2.2 Cerebellum

A statistically significant effect of thiacloprid exposure on *Pcna* transcription (*F*(2, 19) = 9.025, *p* = 0.002) was observed. There was a statistically significant increase at the low dose of 0.06 mg/kg/day (*p* = 0.011) compared with the control group, while a statistically significant decrease with the higher dose of 0.6 mg/kg/day (*p* = 0.011) was observed (Figure 5). There was no sex difference or interaction between treatment and sex. No other difference was observed for the other transcripts in the cerebellum (see Table S4 for the statistical results for all 10 genes and Table S5 for Tukey's post hoc test results).

##### 3.2.2.3 Hypothalamus

A statistically significant effect of the treatment with a reduction of nestin (*F*(2, 19) = 11.914, *p* = 0.018), neurogenin (*F*(2, 19) = 8.349, *p* = 0.003), *Bdnf* (*F*(2, 19) = 6.771, *p* = 0.006) and aromatase (*F*(2, 19) = 6.135, *p* = 0.009) transcription was observed. Post hoc analysis showed that both doses of thiacloprid (0.6 and 0.06 mg/kg/day) led to a significant reduction compared with the control group and a significant decrease of the above‐mentioned markers (nestin: *p* > 0.002; neurogenin: *p* < 0.01; *Bdnf*: *p* < 0.023; aromatase: *p* < 0.046). There was a significant main effect of thiacloprid on hypothalamic synaptophysin (*F*(2, 19) = 5.773, *p* = 0.011), with post hoc showing a significant increase in transcription at the lower dose of 0.06 mg/kg/day (*p* = 0.006) as compared with the control group. There was no sex difference or interaction between treatment and sex. No other difference was observed for the other transcripts in the hypothalamus (see Table S4 for the statistical results for all 10 genes and Table S5 for Tukey's post hoc test results).

##### 3.2.2.4 Hippocampus

A significant main effect of thiacloprid with an increase in transcription levels of the neural markers nestin (*F*(2, 18) = 10.308, *p* = 0.001) and neurogenin (*F*(2, 18) = 11.258, *p* = 0.001) was found. Post hoc analysis showed this impact of thiacloprid due to the higher expression level in the group exposed to 0.6 mg/kg/day compared with the control group (nestin: *p* = 0.002; neurogenin: *p* = 0.002) and the group exposed to 0.06 mg/kg/day (nestin: *p* = 0.002; neurogenin: *p* = 0.001; see Figure 5). Similarly, there was a statistically significant increase in the transcription of *Esr1* (*F*(2, 18) = 8.598, *p* = 0.002), *Esr2* (*F*(2, 18) = 9.106, *p* = 0.002), and aromatase (*F*(2, 18) = 7.508, *p* = 0.004), again with the 0.6 mg/kg/day group significantly higher than the control group (*Esr1*: *p* = 0.011; *Esr2*: *p* = 0.012; aromatase: *p* = 0.028) and the 0.06 mg/kg/day group (*Esr1*: *p* = 0.003; *Esr2*: *p* = 0.002; aromatase: *p* = 0.004) (Figure 5). There was no sex difference or interaction between treatment and sex. No other difference was observed for the other transcripts in the hippocampus (see Table S4 for the statistical results for all 10 genes and Table S5 for Tukey's post hoc test results).

## 4 Discussion

The objectives of the current study were to better define the potential impact of the neonicotinoid thiacloprid on neuroplasticity and the neuroendocrine markers in aquatic (zebrafish) and terrestrial (mice) vertebrates. While low concentrations of thiacloprid did not impact zebrafish eleutheroembryos, neuroplasticity and neuroendocrine biomarkers were impacted in adolescent mice in a dose‐ and sex‐ and region‐dependent manner.

### 4.1 The Effects of Developmental Exposure to Thiacloprid on Zebrafish

Our results show that 4 or 5 days of exposure of zebrafish eleutheroembryos to three different low concentrations of thiacloprid did not affect the mortality or gene transcription in the whole heads. The brain aromatase promoter activity was not affected either. Partial protection was likely conferred by the chorion during the initial 48 h of exposure. However, thiacloprid is expected to readily diffuse into the brain due to the absence of a functional blood–brain barrier at early developmental stages (Jeong et al. 2008), coupled with its moderate lipophilicity (LogP = 1.26) and low molecular weight (252 g/mol). Brain concentrations of thiacloprid were not quantified in the current study, leaving open the question of whether the lack of observed effects reflects limited or absent target‐site exposure, or alternatively, a low sensitivity of nicotinic receptors to thiacloprid under our experimental conditions. Previous studies suggest that neonicotinoid exposure impacts the early developmental stage of zebrafish. High concentrations of neonicotinoids, including thiacloprid, have significant deleterious effects on zebrafish, such as teratogenic effects, heart rate modulation, increased DNA damage, and oxidative stress (Yan et al. 2016; Shukla et al. 2017; Ge et al. 2015; Tian et al. 2020; Aydin 2011; Xu et al. 2022), endocrine‐disrupting effects (Ma et al. 2022), and neurobehavioral consequences (Toğay et al. 2021; Liu et al. 2018; Ma et al. 2019; Xie et al. 2022; Von Hellfeld et al. 2022; Özdemir et al. 2014; Osterauer and Köhler 2008). Early exposure to 45‐ or 60‐mM imidacloprid reduced swimming activity and increased startle response in juvenile and adult zebrafish (Crosby et al. 2015). Similarly, lower concentrations (0.5 mM) of imidacloprid as well as thiacloprid acutely reduced locomotor activity in eleutheroembryos (Könemann et al. 2022), but these deleterious effects were reversible, independent of the window of exposure (Sánchez‐Bayo and Hyne 2014; von Wyl et al. 2023). It was found that concentrations above 100 μg/L (~0.4 μM) affected locomotion and gene transcription linked to neurotransmitter systems (acetylcholine, but also GABA and 5‐HT) (Xie et al. 2022). It should be noted that the sensitivity to neonicotinoids depends on the strain of zebrafish, but to our knowledge, the mechanisms underlying these differences are unknown (Crosby et al. 2015; Vignet et al. 2013). In general, zebrafish brains are known to express eight nAChR subunits (α2, α3, α4, α6, α7, β2, β3, and β4) (Ackerman et al. 2009; Zirger et al. 2003), but the potential direct interaction of thiacloprid or other neonicotinoids with these subunits, in various combinations, has not been characterized to our knowledge. Furthermore, the exact distribution of these subunits, sensitivity as well as their expression level, in various environmental conditions, has not been fully explored. It should be mentioned that the concentrations used in the majority of studies, including ours, are much above environmental concentrations found in aquatic habitats (11.493 ± 5.095 ng L^−1^ or approximately 0.05 nM; Wu et al. 2022; Wang et al. 2023) albeit higher concentrations can be observed locally (up to 1.4 μg/L, or 5 nM when measured) and acute, transient, and local environmental exposure at much higher concentrations cannot be ruled out. Probably, the exposure period of 6 days was not sufficient to induce changes, or the targeted approach may not have covered all potential candidates that thiacloprid could have affected.

It is important to emphasize again that whole heads were investigated in our study for the numerous neuroplasticity, neurogenesis, and neuroendocrine pathways, while a more focused investigation in defined brain regions might have shown different expression patterns. Indeed, as shown below in mice, thiacloprid exposure led to region‐specific alterations in the expression of markers such as *Dcx*, with increased levels observed in the amygdala and decreased levels in the hippocampus. Such opposing effects may have masked any net change if analyses were conducted at the whole‐brain level. Future studies should therefore aim to delineate the region‐specific neurobiological consequences of thiacloprid exposure.

### 4.2 The Effects of Gestational Exposure to Thiacloprid on Mice

In addition to zebrafish, we investigated the impact of developmental exposure to three doses of thiacloprid on female and male mouse offspring during the juvenile stage, focusing on several brain regions important for behavior, including cognition, social interactions, and emotion. The cholinergic system, and more precisely the nicotinic receptors, are already functional as early as gestational day 10 in the mouse cortex and day 11 in the mesencephalon (Atluri et al. 2001). Several studies investigating the impact of early cholinergic alteration, including exposure to nicotine or acetylcholinesterase inhibitors such as organophosphate or carbamate, have shown long‐term alteration of neurobehavioral outcomes (Antonangeli et al. 2023; Muñoz‐Quezada et al. 2013; Bjørling‐Poulsen et al. 2008; Burke et al. 2017; Smith et al. 2010; Castro et al. 2023). We show here, in support of other studies, that thiacloprid impacts the development of the central nervous system of mice, and these alterations are not reversed in the juvenile stage. More importantly, we are the first to show that prenatal exposure to low doses of thiacloprid specifically impacts neurogenesis, neuroplasticity, and neuroendocrine functions in a dose‐dependent and region‐dependent manner.

#### 4.2.1 Effect of Thiacloprid on Neurogenesis

Neurogenesis in mammals is predominant during development but is also observed during adolescence and even later in adulthood in the mammalian dentate gyrus in the hippocampus, as well as in the subventricular zone. Recent studies suggest the possibility of postnatal neurogenesis in additional brain regions such as the amygdala and hypothalamus in mammals (Mohr et al. 2022; Batailler et al. 2014), including some evidence in humans, although this remains a matter of debate (see, e.g., Roeder et al. 2022; Terreros‐Roncal et al. 2022). We found here that in utero exposure to various doses of thiacloprid modulates the transcription of biomarkers of neurogenesis, including *Pcna* (proliferation), nestin (neural progenitor), neurogenin (neuronal specification), and *Dcx* (immature neuron), in both male and female mice later during adolescence. It is well known that the cholinergic system is one of the many neurotransmitter systems regulating neurogenesis, both during development as well as in adults (review in Campbell et al. 2011; Bruel‐Jungerman et al. 2011; Madrid et al. 2021). Previous studies have shown that neonicotinoids can impact neurogenesis in the neonatal cortex, cerebellum, or hippocampus (Sheets et al. 2016; Kimura‐Kuroda et al. 2012; Liu et al. 2016; Singh et al. 2015; Kagawa and Nagao 2018), often at relatively high doses, and likely via the activation of the brain nicotinic receptors. For example, previous studies have shown that nAChR α7 and β2 subunits with clothianidin binding affinity were seen in the dentate gyrus neural progenitor cells (Kaneko et al. 2006), and stimulation of α7 nAChR using nicotine‐cultured hippocampal cells activated ERK 1/2, which promotes the proliferation of neural progenitor cells (Dajas‐Bailador et al. 2002). However, there is little information on the long‐term impact of early cholinergic alteration by neonicotinoids on postnatal neurogenesis.

A few studies suggest that the impact of early exposure to molecules such as chlorpyrifos (Wang et al. 2013) or neonicotinoids, including thiacloprid (Könemann et al. 2022), on neurobehavioral parameters, including neurogenesis, is transient, while other reports suggest otherwise (e.g., Burke et al. 2018; Maeda et al. 2021). Our data show that early thiacloprid exposure will affect markers of neurogenesis in various brain regions later in life, like what was found for nicotine (Liu et al. 2019). The biological mechanism linking early exposure to later neurogenesis was not investigated, but we can hypothesize that early exposure to thiacloprid affects the neural progenitor pools and/or their local environment (stem cell niche; Sharma 2013; Takahashi 2021). The most intriguing observation is the up‐ or down‐regulation of several markers such as PCNA, Neurogenin, or Nestin, depending on the brain region, but also depending on the dose of prenatal exposure. A non‐monotonic dose response is not unusual and was previously described in insects (Baines et al. 2017). These findings, including ours, suggest non‐monotonicity‐like phenomena, but no studies to our knowledge have clear‐cut U‐shaped or inverted U‐shaped toxicity curves. These nonlinear responses may arise from differential binding affinities and activation profiles of distinct nicotinic acetylcholine receptor (nAChR) subtypes, resulting from variable α and β subunit compositions. We cannot exclude that the activation of certain subunits at low doses might lead to a specific transcriptional and physiological outcome, while higher doses could activate different subunits and lead to a different response (Chavez‐Noriega et al. 1997). In addition, we cannot exclude an indirect effect of thiacloprid, affecting notably reactive oxygen species (ROS) and mitochondria (Zouaoui and Rouabhi 2024; Cheng et al. 2025) but also acting as an endocrine disruptor (see below). Similar mechanisms could explain the regional difference observed here and below, given the heterogeneous expression of nAChR subunits across different brain regions. Additional work is still needed in vertebrates, especially systematic, dose‐ranging studies designed to detect how dose/concentration can impact physiology. In addition, future investigations at the cellular level should define how in utero neonicotinoid exposure affects adolescent and adult neurogenesis, in both males and females.

#### 4.2.2 Effect of Thiacloprid on Synapses

In addition to an impact on neurogenesis, thiacloprid also affected the transcription of the synaptic markers synaptophysin and synapsin IIa in the hypothalamus and the hippocampus in a dose‐dependent manner. It is important to keep in mind that these two synaptic markers are not unique to the cholinergic system. While synaptophysin is present in most synaptic vesicles of all neurons (DeLellis and Shin 2006; Kokotos et al. 2019), synapsin IIa is preferentially associated with excitatory neurotransmission and could be involved in maintaining the reserve pool of glutamatergic vesicles (Gitler et al. 2008). These two markers are commonly used as a signature of the potential impact of the chemical as well as the social environment on communication pathways within the central nervous system (Pawluski et al. 2020; Dechartres et al. 2019). In the present work, the impact of thiacloprid on these markers strongly suggests that not only will the cholinergic system be affected, but other cell‐signaling pathways, such as glutamatergic neurotransmission, might also be altered. Treatment with acetamiprid was shown to significantly reduce the levels of glutamate and its *N*‐methyl‐d‐aspartate (NMDA)–like receptor subunits, which could translate into significant memory deficits (Shamsi et al. 2021). The adverse effects of neonicotinoids on neurotransmission depend on the receptors that are activated as well. Clothianidin led to striatal dopamine release via a vesicular‐and calcium‐dependent mechanism that required the activation of α4 or α7 subunits of nAChRs and not the β2 subunit (Faro et al. 2019). Imidacloprid facilitated tyrosine hydroxylase transcription by acting as a partial agonist at α3β4 and α7 receptors, causing long‐term activation of second messenger systems (CREB‐PKA‐ERK and Rho cascade) (Kawahata and Yamakuni 2018). In addition, metabolites derived from thiacloprid that were not investigated in the current study could also have caused the observed effects. Some studies have already supported metabolites as a possible explanation for the toxicity of neonicotinoids (Li et al. 2020; Campbell et al. 2022; Passoni et al. 2021; Caron‐Beaudoin et al. 2017) and for observed sex differences (Kubo et al. 2022). The authors also noted the dependence on the activation of muscarinic acetylcholine receptors (mAChRs) (Cimino et al. 2017; Faro et al. 2019). Future studies should investigate the impact of neonicotinoids on other neurotransmitter systems in detail.

#### 4.2.3 Effect of Thiacloprid on Neuroendocrine Markers

While thiacloprid, like other neonicotinoids, is classically not considered an endocrine disruptor, we observed that several markers linked to steroid action, including estrogen receptors (*Esr1* and *Esr2*) and aromatase transcripts, were also specifically affected by thiacloprid in various brain regions, including the hypothalamus, amygdala, and hippocampus.

Sex steroid hormones such as estrogens have a strong impact on neuronal and glial structure and neurophysiology, in both males and females, and all vertebrate species studied to date. Estrogen receptors alpha and beta are strongly expressed in various brain regions, especially in the hypothalamus and amygdala, but also in the hippocampus (Mitra et al. 2003; Zhang et al. 2002; González et al. 2007; Shughrue and Merchenthaler 2000). Estrogens are classically recognized to be synthesized in the ovaries and placenta, but the brain itself can produce its estrogens by the action of the enzyme aromatase, either from circulating androgens from the gonads or the adrenals (via the presence of aromatase in several regions of the mammalian brain) or from de novo synthesis from cholesterol (e.g., Brandt et al. 2020; Charlier et al. 2010; Schmidt et al. 2008; Charlier et al. 2013). Any alteration of the steroid signaling via disruption of estrogen synthesis or steroid receptor activity will affect neurobehavioral and/or cognitive outcomes (Patisaul 2021; Özel and Rüegg 2023). Previous in vitro work showed that thiacloprid and imidacloprid induced estrogenic activity at high concentrations (> 10 μM) in estrogenic reporter cells (MCF‐7 derived MELN cell line and CHO) (Zhang et al. 2020; Kojima et al. 2004) while other studies with thiacloprid and other neonicotinoids did not induce any estrogenic activity (Westlund and Yargeau 2017; Gea et al. 2022). In addition, neonicotinoids were previously shown to impact steroidogenesis both in vitro and in vivo. Rabbits treated with thiacloprid had a significant decrease in serum levels of gonadal hormones, suggesting an impact of neonicotinoids on steroidogenic enzymes (Islam 2022). Indeed, imidacloprid was shown to disrupt steroidogenesis in an in silico study by impairing the activities of the cytochrome P450 (CYP) enzymes that play a key role in steroidogenesis and steroid catabolism (Bhaskar et al. 2014). Imidacloprid was shown to interrupt steroidogenesis by inhibiting 3β‐HSD and 17β‐HSD (HSD‐hydroxysteroid dehydrogenase) enzyme activities (Lonare et al. 2016). Furthermore, peripheral aromatase expression and activity were shown to be affected by thiacloprid and other neonicotinoids in human H295R adrenocortical carcinoma cells alone (Caron‐Beaudoin et al. 2016) or in coculture models of fetoplacental steroidogenesis of H295R and BeWo cells (Caron‐Beaudoin et al. 2017) by thiacloprid and thiamethoxam, leading to a significant impact on estradiol and estrone production (Caron‐Beaudoin et al. 2018). The impact of neonicotinoids on aromatase is probably due to the activation of nAChRs, as nicotine exposure also leads to a significant reduction of aromatase (von Ziegler et al. 1991; Merii et al. 2022). The observed alterations in estrogen‐related gene expression may reflect a disruption of neurosteroidogenesis, in addition to, or rather than, systemic endocrine disturbances. In particular, the dose‐ and region‐specific modulation of brain aromatase transcription observed in this study is intriguing. The regulation of aromatase expression and activity within the brain is known to be highly region‐specific and sensitive to a range of neuromodulatory signals, including cholinergic input (Li et al. 2018). Indeed, previous studies have documented regional heterogeneity in the expression and control of brain aromatase, with the evidence that compounds like nicotine can differentially affect aromatase activity depending on the brain region (e.g., Konkle and McCarthy 2011; Munetsuna et al. 2009; Biegon 2016). For example, recent human neuroimaging research indicates that nicotine selectively reduces aromatase activity in the thalamus, while leaving hypothalamic and amygdalar enzyme levels largely unaffected (Dubol et al. 2023). This aligns with the notion that local microenvironmental factors, receptor subtype expression, and neural connectivity contribute to region‐dependent regulation of aromatase.

Furthermore, both nicotine and structurally related neonicotinoids have been shown to suppress aromatase activity in various experimental models. This pharmacological overlap lends mechanistic plausibility to the effects observed here and supports the hypothesis that neonicotinoid exposure, such as to thiacloprid, may disrupt local estrogen synthesis within specific brain regions. Such disruptions could have significant implications for neurodevelopment and behavior, given the well‐established roles of brain‐derived estrogens in neurogenesis, synaptic plasticity, and sexual differentiation of the brain.

Our observations that the region‐dependent modulation of aromatase transcription, along with changes in *Esr1* and *Esr2*, strongly suggest a very complex interplay between cholinergic impact, neuroendocrine effects, and consequences on neuroplasticity, including neurogenesis. Indeed, we cannot exclude that some of the effects of thiacloprid on neuroplasticity are not linked to a direct nicotinic activation but are a consequence of local changes in estrogen synthesis and action on the receptor, leading to a disruption of the neurosteroids pathway in various brain regions and broadening the perspective beyond classical endocrine disruption.

These results strongly indicate the need for further work in this area using in vitro and in vivo models to decipher the potential impacts and mode of action of neonicotinoids on the neuroendocrine system. Furthermore, we also need to investigate the impact of thiacloprid and neonicotinoids in general, on neuroplasticity and brain steroid signaling to define if these two aspects are independent or are causally linked. This concept needs to be extended to a much broader global mode of action. Indeed, the observed impact of thiacloprid in one brain region might stem from the modulation of either a very small, well‐defined brain nucleus within that region, or might result from an impact in a different brain area but connected to the area under study. In addition, the brain, while controlling the physiology of each system and organ in an organism, is itself under the influence of various systems, including the immune system and endocrine system, but also by important axes such as the gut–brain axis, the muscle–brain axis, and the liver–brain axis to name a few. Therefore, the modulation of cholinergic‐dependent responses in various peripheral tissues will not only affect the targeted peripheral organ but is also likely to influence brain physiology.

#### 4.2.4 Sex Differences in Thiacloprid Exposure in Mice

We also observed that the highest dose of thiacloprid in our experiment induced a stronger reduction of *Bdnf* and Nestin in the hypothalamus and *Pcna* in the hippocampus in females, while the males were less affected. The implication of biological sex on physiological responses to chemical exposure is relatively common, albeit not very often studied in toxicology, and males are usually more sensitive to environmental stress (Stinson 1985; Assari and Lankarani 2016; Pérez‐Cerezales et al. 2018). The activity of the cholinergic system is partly sexually differentiated, notably through the regulation by circulating estrogens, leading to sex differences in the incidence of disorders such as Alzheimer's disease and nicotine addiction (Russell et al. 2019; Newhouse and Dumas 2015). Human PET imaging revealed that the binding level of α4β2 nAChRs was higher in all brain regions in women than in men (Mukherjee et al. 2018). In rats and mice, the basal expression of α4β2 nAChRs is also higher in most brain regions in females, while repeated nicotine treatment reverses this expression pattern, leading to a higher expression in nicotine‐exposed males compared with females (Koylu et al. 1997; Mochizuki et al. 1998). The sex differences in the cholinergic system likely explains the higher sensitivity of females to a high dose of neonicotinoid observed in the present report and data obtained from other labs, where sex was used as a biological variable (SABV). A few studies showed a higher sensitivity in males. For example, a single intraperitoneal (i.p.) dose of 337 mg/kg imidacloprid to pregnant Sprague–Dawley rats on gestation day (GD) 9 increased plasma cholinesterase activity in male offspring only, and brain region‐specific acetylcholinesterase activity in F1 males and females was observed (Abou‐Donia et al. 2008). Acetamiprid administered by gavage (45 mg/kg/day) from GD 6 to postnatal Day (PND) 21 decreased the acoustic startle response in F1 males and was associated with a marginally significant increase in the number of errors in the Biel maze just after weaning, while leaving the females unaffected (Sheets et al. 2016). Gestational exposure to acetamiprid showed that males in the low‐dose group (1 mg/kg) had a significant increase in sexual and aggressive behaviors, and both low‐ and high‐dose (10 mg/kg) group males showed a significant reduction of anxiety levels during light–dark transition test, while females remained unaffected (Sano et al. 2016). Similarly, imidacloprid (Burke et al. 2018; Saito et al. 2023), dinotefuran (Yoneda et al. 2018), and clothianidin (Tanaka 2012; Kaku et al. 2024) exposure led to sex‐specific changes with elevated motor activity in treated male mice. However, 5 or 50 mg/kg clothianidin (Kubo et al. 2022) decreased locomotor activities, elevated anxiety‐like behaviors, impaired short‐ and long‐term learning memory, increased c‐fos positive cells in the paraventricular thalamic nucleus and the dentate gyrus of the hippocampus in males, potentially due to a sex difference in the pharmacokinetics as higher concentrations of clothianidin and metabolites in blood and urine were found in males. On the other hand, other studies have highlighted a higher sensitivity of females. Gestational exposure to imidacloprid (750 ppm) led to a slight but significant reduction of caudate‐putamen width in F1 female rats on PND 72. Similarly, increased thickness of the hippocampal gyrus and cerebellum height on PND 11 and decreased thickness of the hippocampal gyrus were observed in females following gestational clothianidin exposure (1750 ppm) (Sheets et al. 2016). Nicotine exposure was shown to decrease the expression of the steroidogenic acute regulatory protein (StAR) in CA1, CA3, and dentate gyrus regions of the hippocampus in female rats compared with the control group and male rats (Zhang et al. 2019). Altogether, this data highlights the need to look for potential sex differences in the impact of neonicotinoids on brain structure and physiology. The precise mechanisms remain unclear but are likely influenced by differences in hormone exposure, both during developmental stages (organizational effects) and puberty (activation effects). Additionally, sex‐specific variations in local neurosteroidogenic pathways could contribute to these differences (King and Stocco 2011). Notably, the function and impact of locally produced estrogens may differ between males and females (Cornil 2018) and may be partly independent of gonadal steroid production. The current findings indicate that thiacloprid treatment affects the transcription of aromatase and estrogen receptors. Although the pattern of regulation appears similar in both sexes, the downstream effects on other aspects of neuroplasticity may still diverge between males and females. In addition to the potential sexually differentiated and region‐dependent brain sensitivity, it is important to note that toxicokinetic parameters (absorption–distribution–metabolism–excretion: ADME) are also impacted by sex and gender (Soldin and Mattison 2009) and require to be taken into consideration when analyzing the potential impact of not only neonicotinoids but other chemicals and substances.

## 5 Conclusions

Perinatal exposure to thiacloprid resulted in a dose‐and sex‐dependent alteration of various neuroplasticity and neuroendocrine pathways in specific brain areas in juvenile mice, but not in zebrafish in our experimental conditions. The results show the persistence of long‐term adverse neuroplastic effects after perinatal exposure to toxic chemicals in mice. The results emphasize the difficulty in defining a unique and appropriate model in toxicology studies. Zebrafish eleutheroembryos were continuously and directly exposed to thiacloprid via aqueous immersion, whereas mice were exposed in utero following maternal oral administration. The pronounced toxicokinetic differences between these models—including distinct routes of absorption (transdermal and chorion in zebrafish versus intestinal absorption in mice), distribution patterns (notably divergent plasma protein profiles, particularly albumin levels), and metabolic capacity (reflecting species‐ and age‐specific activity of hepatic enzymes such as cytochrome P450s)—are likely contributors to the observed disparities in thiacloprid effects. These factors warrant further systematic investigation to fully understand how thiacloprid and neonicotinoids in general could impact nontarget species.

More importantly, the cholinergic system in the developing brain of rodents and zebrafish, including analysis of receptor subunits, location, and distribution, is yet to be well characterized to better understand the potential impact of neonicotinoids and other environmental chemicals. In addition, a better characterization of toxico‐kinetics and toxico‐dynamics is required that will allow a better understanding of potential risks posed by neonicotinoids for wild and domesticated species, but also human health.

## Preprint Declaration

This manuscript is based on a chapter from the dissertation of the first author, which is available in the institutional repository. Kunikullaya Ubrangala, K. (2023). Short‐term impact of anthropogenic environment on neuroplasticity: A study among humans and animals. [Doctoral Thesis, Maastricht University]. Maastricht University. https://doi.org/10.26481/dis.20230705kk.

## Author Contributions

**Kirthana Kunikullaya U** **:** data curation, formal analysis, investigation, software, validation, visualization, writing – original draft, writing – review and editing. **Zuzanna M. Baran** **:** data curation, investigation. **Pascal Coumailleau** **:** investigation, supervision, visualization, writing – review and editing. **Laetitia Guillot** **:** investigation, visualization. **Harry W. M. Steinbusch** **:** supervision, writing – review and editing. **Fatima Smagulova** **:** investigation, supervision, writing – review and editing. **Thierry D. Charlier** **:** conceptualization, data curation, formal analysis, funding acquisition, investigation, methodology, project administration, resources, software, supervision, validation, visualization, writing – original draft, writing – review and editing.

## Conflicts of Interest

The authors declare no conflicts of interest.

## Supporting information


**Table S1:** Oligonucleotide sequences used in real‐time quantitative polymerase chain reaction experiments.
**Table S2:** ANOVA output from SExperiment 2a tested in mice during Experiment 2a (D0 versus D.6) in the amygdala, hippocampus, and hypothalamic regions.
**Table S3:** Tukey's post hoc test Experiment 2a tested in mice during Experiment 2a (D0 versus D.6) in the amygdala, hippocampus, and hypothalamic regions.
**Table S4:** ANOVA Output from Experiment 2b tested among mice during Experiment 2b (D0, D0.06, D0.6) in the amygdala, hippocampus, hypothalamus, and cerebellar regions.
**Table S5:** Tukey's post hoc test Experiment 2b tested among mice during Experiment 2b (D0, D0.06, D0.6) in the amygdala, hippocampus, hypothalamus, and cerebellar regions.

## Data Availability

The data that support the findings of this study are available from the corresponding author upon request.
