# Supplementary material for: Perinatal Exposure to the Neonicotinoid Thiacloprid Impacts Transcription of Neuroplasticity and Neuroendocrine Markers in Mice but Not in the Zebrafish Model
Source: J Appl Toxicol. 2025 Aug 3;46(1):179–97. doi: 10.1002/jat.4878 (PMC12668873; doi:10.1002/jat.4878)
Supplement: Supplementary file 1 — Table S1: Oligonucleotide sequences used in real‐time quantitative polymerase chain reaction experiments. Table S2: ANOVA output from SExperiment 2a tested in mice during Experiment 2a (D0 versus D.6) in the amygdala, hippocampus, and hypothalamic regions. Table S3: Tukey's post hoc test Experiment 2a tested in mice during Experiment 2a (D0 versus D.6) in the amygdala, hippocampus, and hypothalamic regions. Table S4: ANOVA Output from Experiment 2b tested among mice during Experiment 2b (D0, D0.06, D0.6) in the amygdala, hippocampus, hypothalamus, and cerebellar regions. Table S5: Tukey's post hoc test Experiment 2b tested among mice during Experiment 2b (D0, D0.06, D0.6) in the amygdala, hippocampus, hypothalamus, and cerebellar regions. [file JAT-46-179-s001.docx]

**The neonicotinoid thiacloprid impacts neuroplasticity and neuroendocrine system differently in mouse and zebrafish models**

**Supplementary Information**

**Supplementary Table 1:** Oligonucleotide sequences used in real-time quantitative polymerase chain reaction experiments

| **Primer name** | **Forward** | **Reverse** |
| --- | --- | --- |
| **Zebrafish** | | |
| elof1 | 5’-AGC AGC AGC TGA GGA GTG AT-3’ | 5’-CCG CAT TTG TAG ATC AGA TGG-3' |
| esr1 (ER Alpha) | 5’-CTG GAG ATG CTG GAC GCT CA-3' | 5’-GCT GCA GCT CCT CCT CCT GG-3’ |
| esr2a (ER Beta2) | 5’-GAT CCT CCT GAA CTC CAA CAT G-3’ | 5’-CCA GCA GAC ACA GCA GCT TGG A-3’ |
| esr2b (ER Beta1) | 5’-GAT CCT GCT CAA CTC TAA TAA C-3’ | 5’-CCA GCA GAT TCA GCA CCT TCC C-3’ |
| cyp19a1b (Aromatase) | 5’-TCG GCA CGG CGT GCA ACT AC-3’ | 5’-CAT ACC TAT GCA TTG CAG ACC-3’ |
| Nestin | 5’-ATG CTG GAG AAA CAT GCC ATG CAG-3’ | 5’-AGG GTG TTT ACT TGG GCC TGA AA-3’ |
| Neurogenin 1 | 5′-TGC ACA ACC TTA ACG ACG CAT TGG-3′ | 5′-TGC CCA GAT GTA GTT GTG AGC GAA-3′ |
| BDNF | 5′-TTA CGA GAC CAA ATG CAA CC 3′ | 5′-CAC GTA AGA CTG GGT TGT CC-3′ |
| Pcna | 5’-CTCACAGACCAGCAACGTCG-3’ | 5’-GGACAGAGGAGTGGCTTTGG-3’ |
| Synapsin IIA | 5’-GTG ACC ATG CCA GCA TTT-3’ | 5’-TGG TTC TCC ACT TTC ACC TT-3’ |
| Synaptophysin | 5’- ATG CAA AGA GCT GCA CGA AC -3’ | 5’-CCC TGA GAG CTG GCA TAC TG -3’ |
| Caspase 3/casp3a | 5’- CCG CTG CCC ATC ACT A -3’ | 5’- ATC CCT ACA CGA CCA TCT -3’ |
| **Mice** | | |
| GAPDH | 5’- GCA TGG CCT TCC GTG TTC C-3’ | 5’- ACC ACC CTG TTG CTG TAG CC-3’ |
| Esr1 | 5’- AGG CAA AAG GGA TTC CAG GG-3’ | 5’- TTG CTG AGG CTT CCT CTT GG-3’ |
| Esr2 | 5’- TTT AGC CAC CCA CTG CCA AT-3’ | 5’- CCT TCA CAG GAC CAG ACA CC-3’ |
| Aromatase (Cyp19a1) | 5’- ATG AGG ACA GGC ACC TTG TG-3’ | 5’- GAG GTT CAC GCC ACC TAC TC-3’ |
| Pcna | 5’- GCC AGA CCT CGT TCC TCT TAG-3’ | 5’- CGT GAG ACG AGT CCA TGC TC-3’ |
| Dcx | 5’- GAC CTG ACC CGA TCC TTG TC-3’ | 5’- ACG TTG ACA GAC CAG TTG GG-3’ |
| Synaptophysin | 5’- ATC AAC CCG ATT ACG GGC AG-3’ | 5’- TCT CTT GAG CTC TTG CCC AC-3’ |
| BDNF | 5’- TTG TTT TGT GCC GTT TAC CA-3’ | 5’- GGT AAG AGA GCC AGC CAC TG-3’ |
| Nestin/Nes | 5’- GTG ACC CTT GGG TTA GAG GC-3’ | 5’- AGA GCA CCT GCC TCT TTT GG-3’ |
| Synapsin IIa | 5’- GAG ACC ATC CGG AGC TTG AG-3’ | 5’- TCA AGT CAT GGG ACA TCG CC-3’ |
| Neurogenin 1/Neurog1 | 5’- CGC TTC GCC TAC AAC TAC ATC-3’ | 5’- TAC TGG GGT CAG AGA GTG GGT-3’ |

**Supplementary Table 2: ANOVA output from SExperiment 2a tested in mice during Experiment 2a (D0 versus D.6) in the amygdala, hippocampus, and hypothalamic regions**

| **Markers** |  | **Amygdala** | | **Hypothalamus** | | **Hippocampus** | |
| --- | --- | --- | --- | --- | --- | --- | --- |
|  |  | **F** | **p** | **F** | **p** | **F** | **p** |
| **DCX** | **Sex** | .166 | .687 | .083 | .775 | .251 | .621 |
|  | **Thiacloprid** | **35.806** | **.000** | .418 | .523 | **4.988** | **.036** |
|  | **Sex * Thiacloprid** | 1.610 | .217 | .004 | .949 | .670 | .422 |
| **Neurogenin** | **Sex** | 1.111 | .302 | 4.063 | .057 | .058 | .812 |
|  | **Thiacloprid** | .665 | .423 | .491 | .491 | **10.903** | **.003** |
|  | **Sex * Thiacloprid** | .522 | .477 | .871 | .361 | .437 | .515 |
| **PCNA** | **Sex** | .871 | .359 | 2.238 | .147 | **5.643** | **.025** |
|  | **Thiacloprid** | 3.298 | .081 | 1.814 | .190 | **31.671** | **.000** |
|  | **Sex * Thiacloprid** | .953 | .338 | 1.182 | .287 | 3.008 | .095 |
| **ER Beta** | **Sex** | .273 | .606 | .310 | .582 | .410 | .528 |
|  | **Thiacloprid** | .591 | .449 | **5.041** | **.033** | 1.297 | .265 |
|  | **Sex * Thiacloprid** | .135 | .716 | .919 | .347 | .009 | .926 |
| **Aromatase** | **Sex** | .742 | .397 | 3.919 | .059 | .682 | .416 |
|  | **Thiacloprid** | 1.646 | .211 | 2.329 | .140 | **68.360** | **.000** |
|  | **Sex * Thiacloprid** | .423 | .521 | 1.136 | .297 | .234 | .633 |
| **ER Alpha** | **Sex** | .570 | .457 | .443 | .512 | .000 | .994 |
|  | **Thiacloprid** | .971 | .333 | .912 | .349 | 2.207 | .149 |
|  | **Sex * Thiacloprid** | .507 | .483 | .338 | .566 | .130 | .721 |
| **Nestine** | **Sex** | .158 | .694 | 2.402 | .134 | .091 | .765 |
|  | **Thiacloprid** | .207 | .653 | **11.339** | **.002** | **23.648** | **.000** |
|  | **Sex * Thiacloprid** | .204 | .656 | 3.047 | .093 | .949 | .340 |
| **BDNF** | **Sex** | 1.921 | .178 | **4.681** | **.040** | .060 | .809 |
|  | **Thiacloprid** | 1.822 | .189 | 3.744 | .064 | .087 | .770 |
|  | **Sex * Thiacloprid** | 1.543 | .226 | 1.424 | .244 | .124 | .727 |
| **Synapsin IIA** | **Sex** | .130 | .722 | .029 | .866 | .084 | .774 |
|  | **Thiacloprid** | 1.533 | .227 | **6.021** | **.021** | **106.908** | **.000** |
|  | **Sex * Thiacloprid** | 1.557 | .223 | .952 | .338 | .003 | .959 |
| **Synaptophysin** | **Sex** | .423 | .521 | 2.990 | .096 | .007 | .934 |
|  | **Thiacloprid** | .034 | .856 | .012 | .913 | **32.413** | **.000** |
|  | **Sex * Thiacloprid** | .326 | .573 | .072 | .791 | .047 | .830 |

**Supplementary Table 3: Tukey’s post hoc test Experiment 2a tested in mice during Experiment 2a (D0 versus D.6) in the amygdala, hippocampus, and hypothalamic regions**

| **Tukey's** |  |  | **Equal variances** | **Levene's Test for Equality of Variances** | | **t-test for Equality of Means** | | | | | | |
| --- | --- | --- | --- | --- | --- | --- | --- | --- | --- | --- | --- | --- |
|  | **Markers** | **Sex** |  |  |  | **t** | **df** | **Sig. (2-tailed)** | **Mean Difference** | **S. E Difference** | **95% CI of Difference** | |
|  |  |  | **Y/N** | **F** | **Sig.** |  |  |  |  |  | **Lower** | **Upper** |
| **Amygdala** | Dcx | M | Y | 2.546 | .137 | -3.296 | 12 | .006 | -0.57 | 0.17 | -0.94 | -0.19 |
|  |  |  | N |  |  | -3.554 | 11.503 | .004 | -0.57 | 0.16 | -0.92 | -0.22 |
|  | Dcx | F | Y | 3.541 | .084 | -5.188 | 12 | .000 | -0.87 | 0.17 | -1.24 | -0.51 |
|  |  |  | N |  |  | -4.758 | 7.144 | .002 | -0.87 | 0.18 | -1.30 | -0.44 |
|  | Synapsin IIA | F | Y | .451 | .516 | -2.343 | 11 | .039 | -0.23 | 0.10 | -0.45 | -0.01 |
|  |  |  | N |  |  | -2.285 | 9.066 | .048 | -0.23 | 0.10 | -0.46 | 0.00 |
| **Hypothalamus** | Synapsin IIA | M | Y | .385 | .544 | 2.298 | 15 | .036 | 0.22 | 0.10 | 0.02 | 0.43 |
|  |  |  | N |  |  | 2.173 | 10.430 | .054 | 0.22 | 0.10 | 0.00 | 0.45 |
|  | Nestin | F | Y | 3.539 | .087 | 3.734 | 11 | .003 | 0.45 | 0.12 | 0.19 | 0.72 |
|  |  |  | N |  |  | 3.931 | 8.977 | .003 | 0.45 | 0.12 | 0.19 | 0.71 |
|  | Bdnf | F | Y | .206 | .659 | 2.517 | 11 | .029 | 0.36 | 0.14 | 0.04 | 0.67 |
|  |  |  | N |  |  | 2.594 | 10.577 | .026 | 0.36 | 0.14 | 0.05 | 0.66 |
| **Hippocampus** | Neurogenin | F | Y | 1.988 | .189 | 2.923 | 10 | .015 | 0.33 | 0.11 | 0.08 | 0.58 |
|  |  |  | N |  |  | 2.658 | 5.843 | .039 | 0.33 | 0.12 | 0.02 | 0.64 |
|  | Pcna | M | Y | 6.886 | .020 | 2.557 | 14 | .023 | 0.31 | 0.12 | 0.05 | 0.58 |
|  |  |  | N |  |  | 2.818 | 10.957 | .017 | 0.31 | 0.11 | 0.07 | 0.56 |
|  |  | F | Y | .038 | .848 | 5.958 | 12 | .000 | 0.59 | 0.10 | 0.38 | 0.81 |
|  |  |  | N |  |  | 5.916 | 10.624 | .000 | 0.59 | 0.10 | 0.37 | 0.81 |
|  | Aromatase | M | Y | .552 | .469 | 5.834 | 15 | .000 | 0.59 | 0.10 | 0.37 | 0.81 |
|  |  |  | N |  |  | 5.641 | 11.469 | .000 | 0.59 | 0.10 | 0.36 | 0.82 |
|  |  | F | Y | 3.446 | .088 | 5.850 | 12 | .000 | 0.66 | 0.11 | 0.42 | 0.91 |
|  |  |  | N |  |  | 6.546 | 9.634 | .000 | 0.66 | 0.10 | 0.44 | 0.89 |
|  | Nestin | M | Y | .250 | .625 | 3.893 | 14 | .002 | 0.40 | 0.10 | 0.18 | 0.62 |
|  |  |  | N |  |  | 3.840 | 12.305 | .002 | 0.40 | 0.10 | 0.17 | 0.63 |
|  |  | F | Y | .155 | .702 | 3.051 | 10 | .012 | 0.60 | 0.20 | 0.16 | 1.04 |
|  |  |  | N |  |  | 3.229 | 7.086 | .014 | 0.60 | 0.19 | 0.16 | 1.04 |
|  | Synapsin IIA | M | Y | 2.222 | .157 | 6.716 | 15 | .000 | 0.64 | 0.10 | 0.44 | 0.85 |
|  |  |  | N |  |  | 6.082 | 8.650 | .000 | 0.64 | 0.11 | 0.40 | 0.89 |
|  |  | F | Y | 4.926 | .046 | 8.819 | 12 | .000 | 0.65 | 0.07 | 0.49 | 0.81 |
|  |  |  | N |  |  | 9.871 | 9.619 | .000 | 0.65 | 0.07 | 0.50 | 0.80 |
|  | Synaptophysin | M | Y | .141 | .713 | 3.875 | 14 | .002 | 0.44 | 0.11 | 0.20 | 0.69 |
|  |  |  | N |  |  | 3.907 | 13.419 | .002 | 0.44 | 0.11 | 0.20 | 0.69 |
|  |  | F | Y | .053 | .822 | 4.345 | 11 | .001 | 0.48 | 0.11 | 0.24 | 0.72 |
|  |  |  | N |  |  | 4.229 | 7.900 | .003 | 0.48 | 0.11 | 0.22 | 0.74 |

**Supplementary Table 4: ANOVA Output from Experiment 2b tested among mice during Experiment 2b (D0, D0.06, D0.6) in the amygdala, hippocampus, hypothalamus, and cerebellar regions**

|  |  | **Amygdala** | | **Cerebellum** | | **Hypothalamus** | | **Hippocampus** | |
| --- | --- | --- | --- | --- | --- | --- | --- | --- | --- |
|  |  | **F** | **p** | **F** | **p** | **F** | **p** | **F** | **p** |
| **DCX** | **Sex** | .014 | .908 | 1.419 | .248 | .952 | .341 | 1.815 | .195 |
|  | **Thiacloprid** | 4.065 | .034 | 1.491 | .250 | 1.843 | .186 | .589 | .565 |
|  | **Sex * Thiacloprid** | .624 | .546 | .896 | .425 | .402 | .675 | .363 | .701 |
| **Neurogenin** | **Sex** | .435 | .517 | 1.310 | .267 | .008 | .928 | .068 | .798 |
|  | **Thiacloprid** | 1.021 | .379 | 2.124 | .147 | 8.349 | .003 | 11.258 | .001 |
|  | **Sex * Thiacloprid** | .318 | .732 | 1.808 | .191 | .144 | .867 | .046 | .955 |
| **PCNA** | **Sex** | .813 | .379 | 2.424 | .136 | .301 | .590 | 1.188 | .290 |
|  | **Thiacloprid** | 4.441 | .026 | 9.025 | .002 | 1.671 | .215 | .548 | .588 |
|  | **Sex * Thiacloprid** | 1.035 | .374 | .948 | .405 | 1.937 | .171 | .214 | .809 |
| **ER Beta** | **Sex** | .011 | .916 | .157 | .696 | 3.641 | .072 | .048 | .829 |
|  | **Thiacloprid** | 1.661 | .216 | .673 | .522 | .833 | .450 | 9.106 | .002 |
|  | **Sex * Thiacloprid** | .123 | .885 | 1.717 | .206 | .312 | .736 | .152 | .860 |
| **Aromatase** | **Sex** | 1.306 | .267 | .265 | .613 | .000 | .990 | .000 | .990 |
|  | **Thiacloprid** | 4.116 | .033 | 1.032 | .375 | 6.135 | .009 | 7.508 | .004 |
|  | **Sex * Thiacloprid** | .356 | .705 | 1.324 | .289 | .158 | .855 | .084 | .920 |
| **ER Alpha** | **Sex** | .397 | .536 | .012 | .916 | 1.962 | .177 | .029 | .866 |
|  | **Thiacloprid** | 1.174 | .331 | .728 | .496 | .356 | .705 | 8.598 | .002 |
|  | **Sex * Thiacloprid** | .050 | .951 | 1.227 | .315 | .059 | .942 | .036 | .965 |
| **Nestine** | **Sex** | .064 | .804 | .509 | .484 | .418 | .526 | .104 | .751 |
|  | **Thiacloprid** | 3.164 | .065 | 1.413 | .268 | 11.914 | .000 | 10.308 | .001 |
|  | **Sex * Thiacloprid** | .012 | .988 | 1.865 | .182 | .163 | .851 | .072 | .931 |
| **BDNF** | **Sex** | .309 | .585 | .651 | .430 | .120 | .733 | .500 | .489 |
|  | **Thiacloprid** | 1.109 | .350 | .961 | .400 | 6.771 | .006 | 1.072 | .363 |
|  | **Sex * Thiacloprid** | .252 | .779 | 1.986 | .165 | .010 | .990 | .968 | .399 |
| **Synapsin IIA** | **Sex** | .935 | .346 | .196 | .663 | .181 | .675 | 1.528 | .232 |
|  | **Thiacloprid** | 1.782 | .195 | 2.722 | .091 | 1.691 | .211 | .678 | .520 |
|  | **Sex * Thiacloprid** | .574 | .573 | .466 | .634 | 1.043 | .372 | .160 | .854 |
| **Synaptophysin** | **Sex** | .437 | .517 | .397 | .536 | .025 | .876 | 1.003 | .330 |
|  | **Thiacloprid** | 1.942 | .171 | .137 | .873 | 5.773 | .011 | .089 | .916 |
|  | **Sex * Thiacloprid** | 1.857 | .183 | 2.058 | .155 | 1.305 | .294 | .218 | .806 |

**Supplementary Table 5: Tukey’s post hoc test Experiment 2b tested among mice during Experiment 2b (D0, D0.06, D0.6) in the amygdala, hippocampus, hypothalamus, and cerebellar regions**

| **Tukey's** | **Dosage** | | **Mean Difference** | **S. E** | **p** | **95% Confidence Interval** | |
| --- | --- | --- | --- | --- | --- | --- | --- |
| **Markers** |  |  |  |  |  | **Lower Bound** | **Upper Bound** |
| **Amygdala** |  |  |  |  |  |  |  |
| Dcx | 0 | 0.06 | -0.72 | 0.24 | .021 | -1.35 | -0.10 |
| Pcna | 0 | 0.06 | -1.40 | 0.45 | .016 | -2.55 | -0.25 |
| Aromatase | 0 | 0.6 | 0.32 | 0.12 | .042 | 0.01 | 0.62 |
| Cerebellum |  |  |  |  |  |  |  |
| Pcna | 0 | 0.06 | -0.59 | 0.15 | .002 | -0.96 | -0.22 |
|  |  | 0.6 | -0.46 | 0.14 | .011 | -0.82 | -0.10 |
| Hypothalamus |  |  |  |  |  |  |  |
| Neurogenin | 0 | 0.06 | 0.49 | 0.13 | .005 | 0.14 | 0.82 |
|  |  | 0.6 | 0.43 | 0.13 | .010 | 0.10 | 0.75 |
| Aromatase | 0 | 0.06 | 0.60 | 0.18 | .010 | 0.14 | 1.06 |
|  |  | 0.6 | 0.45 | 0.17 | .046 | 0.01 | 0.89 |
| Nestin | 0 | 0.06 | 0.58 | 0.14 | .002 | 0.22 | 0.94 |
|  |  | 0.6 | 0.57 | 0.14 | .001 | 0.22 | 0.91 |
| Bdnf | 0 | 0.06 | 0.53 | 0.16 | .010 | 0.12 | 0.94 |
|  |  | 0.6 | 0.45 | 0.16 | .023 | 0.06 | 0.85 |
| Synaptophysin | 0 | 0.06 | -1.22 | 0.34 | .006 | -2.10 | -0.35 |
| **Hippocampus** |  |  |  |  |  |  |  |
| Neurogenin | 0.6 | 0 | 2.38 | 0.59 | .002 | 0.88 | 3.88 |
|  |  | 0.06 | 2.71 | 0.64 | .001 | 1.09 | 4.34 |
| ER Beta | 0.6 | 0 | 1.30 | 0.40 | .012 | 0.28 | 2.31 |
|  |  | 0.06 | 1.75 | 0.43 | .002 | 0.65 | 2.86 |
| Aromatase | 0.6 | 0 | 1.03 | 0.36 | .028 | 0.11 | 1.95 |
|  |  | 0.06 | 1.48 | 0.39 | .004 | 0.48 | 2.48 |
| ER Alpha | 0.6 | 0 | 1.25 | 0.38 | .011 | 0.28 | 2.23 |
|  |  | 0.06 | 1.64 | 0.42 | .003 | 0.57 | 2.70 |
| Nestin | 0.6 | 0 | 2.12 | 0.54 | .002 | 0.76 | 3.49 |
|  |  | 0.06 | 2.31 | 0.58 | .002 | 0.83 | 3.79 |
